# A Maltese Perspective of Sex: a Maltese cross-sectional study

**DOI:** 10.1093/sexmed/qfae095

**Published:** 2025-02-21

**Authors:** Matthew Bartolo, Vincent Marmara, Danica Cassar, Arianna Miclet

**Affiliations:** Willingness, Haz-Zebbug, Malta, ZBG1511; University of Malta, Department of Business and Enterprise, Msida, Malta, MSD2080; Willingness, Haz-Zebbug, Malta, ZBG1511; Willingness, Haz-Zebbug, Malta, ZBG1511

**Keywords:** sexual health, sexual behavior, cross-sectional study, Malta, sex, sex duration, sex frequency

## Abstract

**Background:**

This research constitutes a pioneering cross-sectional investigation into sexual behaviors within the Maltese population, centered on the examination of meaning of sex, sex frequency, sex duration, sexual satisfaction, and discussions about sex.

**Aim:**

The aim of this study is to explore how the Maltese define the term “sex” and with whom they discuss sexual matters while testing the hypotheses that the frequency and duration of sexual activity, as well as the discussion of sexual matters, influence sexual satisfaction.

**Methods:**

Data were collected via a computer-assisted telephone interview method, using a randomized sample of 400 Maltese adults aged 18 and above. The questionnaire included 33 items addressing various aspects of sexual behavior. The responses were analyzed using SPSS, applying Mann–Whitney *U*, Kruskal–Wallis, and chi-square tests to assess relationships between variables.

**Outcomes:**

To gauge participants’ sexual satisfaction, respondents were tasked with rating their contentment with their sexual lives on a scale from 1 to 5.

**Results:**

Results revealed that 26.9% of respondents defined sex as a “sexual act between two persons,” while 26.8% linked it to “an act of love.” Few participants associated sex with pleasure or intimacy, reflecting possible conservative cultural influences. A majority (61.7%) felt comfortable discussing sexual matters with their partners, while only 26.6% were comfortable doing so with friends. The average sexual frequency was 5.69 times per month. Sexual satisfaction was positively correlated with frequency as individuals reporting more frequent sex were generally more satisfied with their sex lives. However, no significant association was found between the duration of sexual activity and sexual satisfaction.

**Clinical translation:**

The study’s clinical implications provide valuable insights for healthcare professionals, policymakers, and researchers grappling with sexual health concerns within the Maltese population.

**Strengths and limitations:**

This pioneering study provides new insights into sexual behaviors in Malta, with stratified sampling enhancing the sample’s representativeness. However, social desirability and recall bias may affect the accuracy of self-reported data, and the focus on age, gender, and relationship status overlooks other factors, such as cultural or religious influences, that may offer a deeper understanding of sexual behaviors.

**Conclusions:**

The Maltese population demonstrates a diverse understanding of “sex” while also revealing that most individuals feel comfortable discussing sexual matters with partners but not with friends; additionally, the population reports an average sexual frequency of 5 times per month, lasting between 26 and 45 minutes, with the majority expressing overall satisfaction with their sexual lives.

## Introduction

Sexual health and sex satisfaction are important aspects to an adults’ life.^1^ Frequency and quality of sexual intercourse are both positively related to increased happiness.^2^ However, little is known about views on sexual satisfaction and factors that can impact sexual health and satisfaction internationally.^1^

### Meaning of sex and talking about sex

The meaning given to sex varies from 1 individual to another.^3^ Previous research shows that sex was often associated with the concept of love.^4^ However, on what is considered as sex, there is more variability. Generally, people consider penile–vaginal intercourse as “sex,” but when it comes to penile–anal, oral–genital, or manual–genital, the line is more blurred.^5^ Internationally, there is not enough research into understanding what sex means to different populations.

In regards to talking to friends or partners about sexual-related matters, Canadian men report having “guy talks.” They might talk about their sexual activity but not their sexual health.^6^ Further, a study in the United Kingdom found that talking to a family doctor about sexual matters was the most preferred.^7^ It was also found that talking to a family member or friend was also a common way to discuss sex matters, particularly for women, with 41.8% reporting this as a source of help.^7^

### Sexual satisfaction

Sexual satisfaction is an important component to adult’s well-being and sexual health.^1^^,^^8^ Factors such as the couple’s relationship, sex, age, health, communication, religion, culture, and socio-economic status among others can impact sexual satisfaction.^1^^,^^8^ A study looking at adults from the United States found sexual satisfaction peaks in the mid-to-late 30s, while it declines after 70 years of age.^1^ Older participants from Portugal, Denmark, Belgium, and Norway reported that 40%-60% of participants were sexually satisfied.^9^ This study also highlighted that relationship status was a significant predictor of sexual satisfaction, with partnered individuals reporting higher satisfaction levels.^9^ A similar result in Brazil reported that, among older people, around 80% reported feeling sexually satisfied.^10^

Sex satisfaction in Sub-Saharan Africa reports a gender difference with males significantly more satisfied with their sex lives than females.^11^ Further, 88% of men and 76% of women found the sex in a relationship very or extremely physically pleasurable.^12^ Despite all of this information, more needs to be known about sexual satisfaction from an individual’s point of view around the world.^13^

### Frequency of sex

Sexual frequency and duration of sex are both important measures to assess sexual activity and overall sexual well-being.^14^ The former is more frequently used than the latter.

Recent research highlights that sexual frequency in both the United States and Australia is decreasing, especially in younger men and women.^12^^,^^15^^,^^16^ It has been found that the average Australian has a mean of 1.8 times per week,^16^ while in the United States, the average was around at least once weekly or more.^15^

In Brazil, the average frequency of sex per week is 3.1 times for men and 2.8 times for women.^17^ Similarly in Turkey, men have sex around 3-4 times per week, while women have sex twice a week.^18^ In Britain, the median number of sex frequency reported for men was 3 times per month, while for women, it started as 4 times but decreased to 3 per month with time.^19^ Further averages of sex frequency per month for Indians is 7 times; for Bangladeshis, it is 8 times, while for Nepalese, it is 11 times.^20^ When examining sexual frequency, it is notable that longitudinal studies from both the United States^15^ and Britain^19^ indicate a slight increase in sexual inactivity over the years. However, the average sex frequency per month seems to be within the same lines across the countries mentioned.

### Duration of sex

In a US sample, the average duration of sexual activity for women was an average duration of 24.1 minutes and men a slightly longer, an average of 24.8 minutes.^21^ Further, a systematic review found that the duration for healthy participants was around 32.38 minutes.^22^ It was also noted that duration is influenced by various factors, including the couple’s characteristics and age. A negative correlation was found between age and the duration of sexual intercourse, suggesting that younger participants may engage in longer durations.^22^

### Sex within the Maltese islands

Malta is an island located in the centre of the Mediterranean that is both the smallest and most densely populated country in Europe, with a population of approximately half a million residents.^23-25^ The Maltese population has been highly influenced by the Roman Catholic Church when it comes to ways of living.^26^^,^^27^ Consequently, this has impacted sexual practices and attitudes. Over the past decade, there has been a significant shift toward more fluid and open sexual attitudes and behaviors.^27^

From our work conducted in Malta as well as searching databases, very limited research can be found about sex behaviors and attitudes within the Maltese population.^26-28^ Despite finding a few scientific papers and dissertations under the topic of sex or sex education, very little information is available about the topic across the islands.^29^ Further, none of these papers look into gaining a profile of sexual behaviors in Malta.

In order to contribute to this gap, this is the first and largest study of the Maltese population looking at understanding various behaviors and aspects of sex. This study aims to explore the Maltese population’s understanding of the term “sex” and to investigate if the frequency and duration of sexual activity can influence sexual satisfaction. The study will address the following exploratory questions: (1) How do the Maltese define the term “sex”? (2) With whom do the Maltese discuss sexual matters? Additionally, it will test the following hypotheses: (3) Does the frequency of sex impact sexual satisfaction? (4) Does the duration of sex impact sexual satisfaction? (5) Does discussing sex correlate with higher sexual satisfaction?

## Methods

Data were collected in the form of a questionnaire. The items in the questionnaire were designed by the researchers and included 33 items. A multi-disciplinary team composed of sex and relationship therapists, psychologists, sex educators, and gynecologists met up multiple times to discuss and consult the design of the questionnaire and its’ items. All of this information was then passed on to a statistician who designed the questionnaire.

The study included a sample size of 400 among the Maltese population aged 18 and above. Results carry a confidence level of +/-4.9% and 95% confidence level. The sample collected was representative based on age, gender and district using quota sampling. The recruitment was done through random mobile/telephone numbers. The numbers were randomly generated through an MS Excel (Version 2019) random number generator. The team of data collectors had to call a large number of individuals to reach the right quotas to have a representative sample based on the mentioned demographics. However, once individuals accepted to participate, most of the individuals accepted to reply to most of the questions. Hence, minimal missing data were registered for individual questions. Following this, a computer-assisted telephone interview method was followed. A pilot of 20 questionnaires was done prior to collecting the data. No recommendations or changes emerged from the pilot questionnaires. The questionnaire was predominantly administered in Maltese as this was the preferred language for the majority of participants. However, upon request, a minority of participants were provided with the questionnaire in English, and their responses were collected in English.

The data were collected between July and September 2022. Upon being contacted, the purpose of the study was explained to participants verbally. Verbal consent was obtained from each participant before collecting any information. For participants who did not consent or wish to participate, the phone call was ended and no data were collected. The data collected was fully anonymized, and no identifier was required.

The data were coded and analyzed in IBM SPSS Statistics v.20. A 0.05 level of significance was used when applying hypothesis tests. Primarily, a Shapiro–Wilk test was applied to test whether the continuous variables follow the normal distribution, from which we concluded that non-parametric tests will be applied throughout the analysis. The Mann–Whitney *U* test was used when comparing 2 categories, and the Kruskal–Wallis test was used when comparing more than 2 categories. Furthermore, the chi-square test was used to test for association between 2 categorical variables.

## Results

The demographic data of the participants can be found in Table 1.

**Table 1:** Participant characteristics.

**Demographics**	**Percentage (n = 400)**
Gender	
Male	49
Female	51
Age (years)	
18-25	13
26-35	19
36-45	16
46-55	15
56-65	15
65+	22
Sexual orientation	
Heterosexual	96
Homosexual	1.3
Bisexual	2.7
Civil status	
Single	13.2
Married	53.8
In a relationship	23.1
Separated/Divorced/Annulled	6.1
Widowed	3.9

### The meaning of the word “sex”

Through the use of an open question, participants were asked what they understood by the word “sex.” The top 5 answers were “sexual act between two persons” (26.9%), “an act of love” (26.8%), “gender” (17.5%), “sexual act” (12.1%) and “sexual act between male and female” (9.3%). Only 28 participants associated intimacy with sex, while only 20 participants associated it with pleasure. Surprisingly, only 2 participants have mentioned that they “don’t know” what sex is. Table 2 presents the results of what the participants understood from the word sex according to their civil status.

**Table 2:** What the participants understand by the word sex and their civil status.

**Understanding of sex**	**Single** **(%)**	**Married** **(%)**	**In a relationship** **(%)**	**Separated/Divorced/Annulled (%)**	**Widowed** **(%)**
Sexual act between 2 persons	11.5	21.1	40.1	44.4	34.8
An act of love	26.9	34.6	15.3	5.6	0.0
Gender identification	6.4	11.9	19.7	0.0	0.0
Sexual act	14.1	7.9	5.8	30.6	0.0
Sexual act between a male and a female	16.7	7.9	5.1	2.8	30.4
Intimacy	9.0	6.9	6.6	8.3	4.3
Pleasure	3.8	2.2	5.8	5.6	0.0
Other	1.3	0.9	0.0	0.0	30.4
Unity	2.6	2.5	0.0	0.0	0.0
Marriage	0.0	2.5	0.0	0.0	0.0
A relationship	5.1	0.9	0.7	0.0	0.0
Reproduction	2.6	0.0	0.7	0.0	0.0
Don’t know	0.0	0.6	0.0	2.8	0.0

### Talking about sex

Most participants (61.7%) reported feeling very comfortable talking about sex with their partner (μ = 4.38 ± 0.08; score range = 1-5) with males (μ = 4.40 ± 0.13) scoring a bit higher than females (μ = 4.37 ± 0.11) (*P*-value = .238). However, when it comes to speaking about sex with their friends, only 26.6% reported feeling very comfortable (μ = 3.37 ± 0.11; score range = 1-5). With regard to talking about sex with peers, females scored statistically significantly higher (μ = 3.48 ± 0.16) than males (μ = 3.26 ± 0.17) (*P*-value = .044). In addition, the youngest participants (μ = 4.00 ± 0.28) are more likely to talk to friends about sex than the eldest group (μ = 2.07 ± 0.22). The likelihood of talking to friends about sex decreases as the age increases (*P*-value <.001). Overall, the Maltese population prefers discussing sex with partners over peers, though this varies by sex and age.

After applying the chi-square test, we found a significant association with those who said that they are “comfortable to talk with their partner about sex” and those who are “happy with their sex life.” In fact, the more the respondents claimed that they are happy to discuss with their partner about sex, the more likely that they are satisfied with their life (*P*-value <.001). Participants (89.6%) who feel very comfortable discussing sex with their partner are satisfied with their sex life. Conversely, 66.7% of those who do not discuss about sex with their partner are still satisfied with their sex life.

### Frequency of sex

The study revealed that the average frequency of sex was 5.69 times per month. Only 6.7% of the sample reported having sex more than 15 times a month, while 16.1% of the participants said that they never have sex. This 16.1% is mainly made up of participants who are widowed and older adults (see Table 3).

**Table 3:** Demographics of participants who do not have sex.

**Demographics**	**Percentage (n = 64)**
Gender	
Male	11.9
Female	20.7
Age (years)	
18-25	0
26-35	4.5
36-45	3.3
46-55	4.9
56-65	18.6
65+	62.5
Civil Status	
Single	27.0
Married	13.5
In a relationship	0.8
Separated/Divorced/Annulled	20.0
Widowed	95.7

Males seem to be slightly more sexually active than women, with men’s average of sexual frequency being 5.90 ± 0.64 times and women’s average being 5.46 ± 0.69 times (*P*-value = .288). The 18- to 25-year-olds were the most sexually active, while the 66+ year olds were the least sexually active. This result was found to be statistically significant (*P*-value <.001). Table 4 presents the average sexual frequency of the different age ranges.

**Table 4:** The average frequency of sex across gender, age range, and civil status of the different age ranges.

**Demographics**	**Mean (SD)**
Gender	
Male	5.90 (5.49)
Female	5.46 (5.59)
Age (years)	
18-25	8.73 (6.83)
26-35	7.32 (5.70)
36-45	7.54 (5.71)
46-55	6.21 (4.55)
56-65	4.33 (3.82)
65+	0.76 (1.07)
Civil status	
Single	4.03 (6.24)
Married	5.57 (4.94)
In a relationship	8.74 (5.70)
Separated/Divorced/Annulled	2.97 (4.08)
Widowed	0.00 (0.00)

The results suggest that individuals who were in a relationship but not married were the most sexually active, while those who were widowed were the least sexually active (*P*-value <.001). Table 4 presents the average times of sexual frequency of individuals based on their gender, age range, and civil status.

The more satisfied the participants were with their sexual life, the more frequency of sex they reported (*P*-value <.001) (see Table 5). Participants were also asked if they desired more sex, with 63.1% responding no, indicating that most do not wish to increase their sexual activity. However, 88.1% of those dissatisfied with their sexual life expressed a desire for more sex (*P*-value <.001).

**Table 5:** The average frequency of sex based on the participants’ level of sexual satisfaction.

**Sexual satisfaction**	**Mean (SD)**
Not satisfied at all	0.53 (1.06)
Not satisfied	0.81 (1.11)
Neutral	2.76 (3.69)
Satisfied	5.34 (4.22)
Very satisfied	7.89 (6.30)

### Duration of sex

Majority of participants said that they take between 26 and 45 minutes to have sex (39.3%). The average time is 27 minutes. Males seem to last longer (28 minutes) than females (25 minutes) (*P*-value = .185). In addition, those who are more sexually satisfied on average last 27 minutes, while those who are not sexually satisfied at all last on average around 15 minutes (*P*-value = .428). Having said this, none of these latter results were found to be statistically significant.

The youngest participants take around 33 minutes, while the older participants take around 26 minutes (*P*-value <.001). In addition, individuals who are single take around 35 minutes, those who are in a relationship take around 29 minutes, and married individuals take around 24 minutes (*P*-value <.001).

### Sexual satisfaction

A total of 44% of participants reported being very satisfied with their sexual life, while 34.1% indicated that they were satisfied. Females seem to be slightly more satisfied with their sexual life (μ = 4.15 ± 0.12) than men (μ = 4.09 ± 0.14) (*P*-value = .681). Only 2.9% were not satisfied at all with their sexual life, and 4.7% were not satisfied. Eighteen- to 25-year-olds seemed to be most satisfied with their sex life (μ = 4.37 ± 0.88), while 65+ year olds seemed to be the least satisfied (μ = 3.53 ± 1.21). This was found to be statistically significant (*P*-value <.001). Table 6 presents sexual satisfaction across gender, age range, and civil status.

**Table 6:** Sexual satisfaction across age range, gender, and civil status.

**Demographics**	**Mean (SD)**
Gender	
Male	4.09 (1.05)
Female	4.15 (0.97)
Age (years)	
18-25	4.37 (0.88)
26-35	4.29 (0.80)
36-45	4.17 (1.03)
46-55	4.22 (0.86)
56-65	4.20 (0.99)
65+	3.53 (1.21)
Civil status	
Single	3.59 (1.13)
Married	4.25 (0.85)
In a relationship	4.43 (0.80)
Separated/Divorced/Annulled	3.81 (1.04)
Widowed	2.87 (1.66)

## Discussion

All exploratory questions and hypotheses posited for this study have been answered thoroughly.

## Meaning of sex

The most common response to the exploratory question “How do the Maltese define the term ‘sex’?” was a “sexual act between 2 persons.” This can suggest that many Maltese may have an inclusive view of sex as they did not specify the genders involved, which could imply a more accepting attitude toward homosexual relationships. However, 9.3% of respondents explicitly defined sex as a “sexual act between a male and a female,” indicating some adherence to traditional gender norms.

Interestingly, the general emphasis on “2 persons” suggests that most respondents conceptualize sex as an activity limited to 2 individuals, excluding practices such as threesomes or orgies. Further, this response may also imply that masturbation is not commonly regarded as “sex” by the Maltese. Furthermore, few participants described sex in terms of “intimacy” or “pleasure,” which contrasts with the broader societal shift from seeing sex primarily as reproductive to viewing it as a form of social interaction.

In addition, some of the current study’s participants also defined sex as “an act of love.” This aligns with previous research that found a common association between sex and the concept of love.^4^ This divergence could be influenced by the significant cultural and moral authority of the Catholic Church in Malta.^26^^,^^27^ Additionally, in a qualitative study, some individuals considered sex as a broad term that constituted a variety of individual acts; sex is not only about sexual intercourse but also other factors such as intimacy and closeness with one’s partner, which are more important when it comes to defining sex.^30^

A considerable number of people reported that “sex” meant “gender identification,” which implies that these individuals might engage in sex to explore their gender. Hence, this also implies that a substantial number of people question their gender identity. Ventriglio and Bhugra highlighted gender as a societal role and a means for individuals to express themselves; they also emphasize that individuals define their gender through acts.^31^ Furthermore, the fact that only 2 participants stated that they did not know what sex is can suggest that sexual education in Malta may be contributing positively to public understanding of the topic.^26^ Alternatively, it is possible that these 2 participants, who belong to either the married or separated/divorced/annulled groups, may have been uncomfortable discussing their personal definition of sex, which could explain their response.

Overall, it was difficult to compare what the Maltese understood with the word “sex” with other populations due to the very little research existing on the topic. This has already been highlighted within research over the years.^5^

### Talking about sex

In exploring the question “with whom do the Maltese discuss sexual matters?”, this study focused only on discussions with partners and peers. It was found that the majority were very comfortable talking about sex with their partner as found by other research.^32^ However, fewer participants were comfortable with talking about sex with their friends, females being slightly more comfortable than males. In fact, in other research, it was found that Canadian men were comfortable with talking about their frequency of sex but not about more sensitive topics on sex.^6^ Another study found that individuals feel the least comfortable to discuss sensitive topics on sexuality with their friends and family members.^7^ The Maltese population’s greater comfort discussing sex with partners rather than friends may stem from the close-knit society, where such topics are typically kept private.^24^ The study, however, did not investigate participants’ comfort levels in discussing sexual matters with medical professionals or other family members, which could have provided additional insights.

In our study, we aimed to test the hypothesis of “Does discussing sex correlate with higher sexual satisfaction?”. From our participants, we could see that individuals who discussed sex with their partners were more likely to be satisfied with their sex life. This means that communication about sexual concerns, needs, and desires can improve sexual satisfaction. This was also highlighted in another study.^33^

Overall, nationwide campaigns about normalizing talking about sex with partners and peers could help improve individuals’ sexual health and well-being.

### Frequency of sex

The Maltese seem to have sex around 5 times per month, which is a little over than once a week. This statistic seems to align with the American^15^ and Australian population.^16^ Unfortunately, limited literature could be found to compare frequency of sex within Europe. A British study found that the average sexual frequency for adults is about 3-4 times per month.^19^ In comparison, Maltese adults may engage in sexual activity 1-2 times more than British adults per month, indicating slightly higher levels of sexual activity in Malta. Further to this study,^19^ the only other research found about sexual activity in Europe was for older adults.^9^ In our study, the majority of older adults barely had sex once a month, while in some other European countries, the average seemed to be 2-3 times a month. Therefore, Maltese older adults seem to be less sexually active than such populations in Norway, Denmark, Belgium, and Portugal.^9^

When comparing sexual frequency across ages further, it was found that the youngest sample was having the most frequent sex, while the eldest was having the least sex. It is well known that sexual frequency is at its highest at the beginning of a relationship, which then leads to a decline.^32^^,^^33^ This variation in sexual activity can be attributed to several factors. Younger individuals often possess higher levels of physical energy and may engage in sexual activity as a form of exploration and self-discovery.^34^^,^^35^ In contrast, older adults may face a decline in sexual activity due to the loss of a partner or age-related physical and mental health issues.^36-38^ Furthermore, a decrease in frequency of sex can be noticed within the groups between 26- and 55-year-olds. Child rearing is 1 of the main activities during this age period according to the family life cycle theory, which could explain the lesser sexual activity due to the roles and responsibilities these bring with it.^39^

This study managed to answer the first hypothesis “Does the frequency of sex impact sexual satisfaction?”. Our results show that in the Maltese population, higher reported sexual frequency is linked to greater sexual satisfaction, with most participants not wanting more sex than they currently have. This is conforming with other findings.^4^^,^^10^ In our sample, females seemed to be slightly more sexually satisfied than men. This could be in line with the research found by McNulty et al., which can be attributed to men prioritizing their partner’s sexual satisfaction more than women.^40^ In women, research shows that the most important predictors for sexual satisfaction include relationship satisfaction and frequency of sexual interactions.^8^

### Duration of sex

Finally, the study explored “Does the duration of sex impact sexual satisfaction?”. While no significant correlation was found between duration of sex and sexual satisfaction, the study could still withdraw information about sex duration. It was found that the average reported duration was 27 minutes, slightly longer than findings from studies in Japan being 13.6-14.5 minutes^41^ and Australia being 8.5-29.5 minutes.^42^ However, the lack of specificity in distinguishing between foreplay and intercourse in our study may have influenced these results, suggesting that future research should focus on refining these measures to better understand the role of duration in sex behaviours and sexual satisfaction.

### Limitations of the study

Whilst our study has contributed to provide insight into the Maltese’s population attitude and behaviour toward sex, this study poses limitations. Firstly, even though the questionnaire was done through phone calls, there still was an element of social desirability bias in the data. This could be due to the negative connotations surrounded around sex that made the participants want to be seen in a positive light.

Secondly, participants were asked to rate the level of sexual satisfaction, which can be subjective. Even though with validated scales, there still can be a level of subjectivity; we suggest that for future research, the New Sexual Satisfaction Scale^43^ would be used to minimize subjectivity as much as possible.

Thirdly, participants were asked about the duration of having sex; in this study, we did not ask participants to distinguish between foreplay and the sexual act, which could have provided different results in terms of duration of sex. Fourthly, the cross-sectional design of the study limits the ability to draw causal inferences as it only captures a snapshot in time rather than examining changes over time. Lastly, the study was conducted in Malta, which may limit the applicability of the results to other regions or cultures*.*

### Future recommendations

As this is the first study of its kind, replication in future years is recommended to analyze shifts in attitudes and behaviors toward sex. This would help identify trends and deepen understanding of factors influencing sexual behavior and perceptions in Malta.

Future research using qualitative research, specifically in-depth interview and/or focus groups, could unravel individual narratives, providing a richer understanding of the observed trends.

The correlation between sexual communication and sexual satisfaction highlights the importance of promoting open conversations about sex within intimate relationships. Clinicians and mental health professionals should encourage individuals and couples to discuss sexual concerns, preferences, and desires as a strategy to enhance their sexual well-being. Further, age-appropriate sex education campaigns should be incorporated into schools to help young people feel more comfortable and open discussing sex with partners and peers from a young age.

The study indicates lower sexual activity among older Maltese adults compared to other European populations. Policymakers could develop and implement nationwide awareness and promotion of sexual health and well-being among older adults to destigmatize the topic. Further, clinicians should receive specialized training on addressing sexual needs and concerns during routine check-ups to help with appropriate referrals for psychosexual education and counseling. Incorporating sexual health discussions into routine healthcare for older adults can help address these concerns and improve sexual activity and satisfaction.

## Conclusions

This study highlights that while the Maltese population generally demonstrates a clear understanding of what constitutes “sex,” their definitions vary, with some adhering to traditional views and others adopting more inclusive perspectives. Additionally, it was found that Maltese individuals feel most comfortable discussing sexual matters with their partners but are significantly less open when discussing such topics with friends, suggesting that sexual communication is largely confined to private settings. Furthermore, the Maltese population have sex around 5 times per month lasting between 26 and 45 minutes with the majority of the Maltese population being sexually satisfied.

In conclusion, this pioneering study lays a solid foundation for subsequent explorations in a society where conservative values have traditionally held sway.
